# Extraosseous Ewing's sarcoma / primitive neuroectodermal tumor of the sacral nerve plexus

**DOI:** 10.4103/0971-3026.50841

**Published:** 2009-05

**Authors:** MK Narula, Nishant Gupta, Rama Anand, Sudhir Kapoor

**Affiliations:** Department of Radiodiagnosis and Sucheta Kriplani and Kalawati Saran Children Hospital, New Delhi-110 001, India; 1 Department of Orthopedics Lady Hardinge Medical College, and Associated Smt. New Delhi-110 001, India

**Keywords:** Extraosseous Ewing's sarcoma, primitive neuroectodermal tumor, sacral nerve plexus

## Abstract

We report an unusual case of Ewing's sarcoma / primitive neuroectodermal tumor (PNET) of the sacral nerve plexus in a 9-year-old boy who presented with a soft tissue swelling and severe piercing pain in the lower back region. MRI of the lumbosacral spine showed a lobulated soft tissue mass with clubbed finger-like projections along the path of the sacral nerves, which had caused widening of the spinal canal and the sacral foramina (S2–S4 level). There was presacral extension and posterior scalloping of the sacral vertebrae. Histopathology of the lesion confirmed Ewing's sarcoma / PNET of the sacral spinal nerve plexus. The patient responded favorably to chemotherapy and radiotherapy, showing clinical and radiological improvement.

Extraosseous Ewing's sarcoma (EES) / primitive neuroectodermal tumor (PNET) of the sacral nerve plexus is an extremely rare primary soft tissue malignancy. It has been grouped along with Ewing's sarcoma of the bone (ESB) under the Ewing's sarcoma family of tumors (ESFTs).[1] Many cases of EES arising in the paravertebral region and epidural space have been reported;[2–4] however, the involvement of the sacral spinal nerve plexus is rarely described in the literature, with only a few cases reported in the world so far.[5] In the only case reported from India, there was involvement of the lumbosacral region in an adult patient.[2] The rarity of this disease in children prompted the description of this case.

## Case Report

A 9-year-old apparently healthy boy presented with a gradually increasing painful swelling of 4 months' duration over his lower back. The pain was localized to the lower back, severe in intensity, and piercing in nature; it did not radiate and increased with movements. It was associated with urinary incontinence for the same duration. There was no history of trauma. There was no history of any significant medical or surgical illness in the child or his family.

Local examination of the site revealed a small tender swelling in the lumbosacral region, with no signs of inflammation, no visible scar / sinus, and no abnormal pulsations. The swelling was fixed to the underlying structures and the overlying skin was freely mobile. The child was anemic, with hemoglobin of 10.2 gm%; the ESR was 62 mm in the first hour (Westergren's method). The other routine investigations were within normal limits.

**Figure 1 (A,B):**
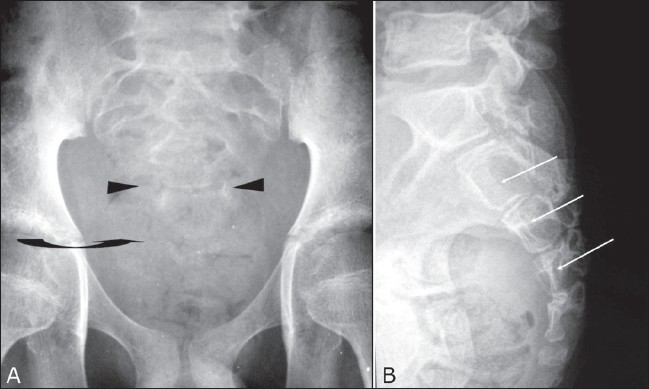
Anteroposterior (A) and lateral (B) plain radiographs of the lumbosacral (LS) spine reveal posterior scalloping of the sacral vertebrae (S2–S4) (arrows), widened and ill-defined sacral foramina (arrowheads), and an overlying right-sided soft tissue haze (curved arrow)

**Figure 2 (A–D):**
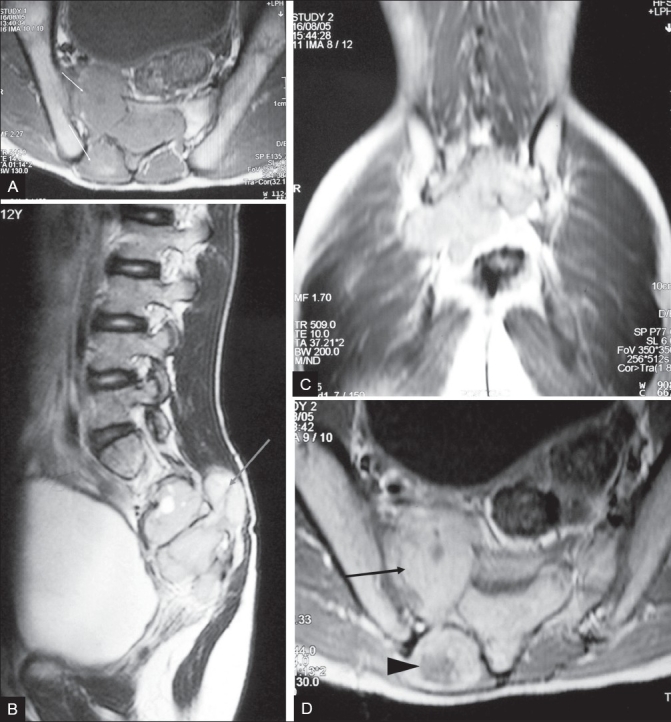
T1W axial (A) and T2W mid-sagittal (B) MRI images of the LS spine show a lobulated soft tissue mass (arrows), isointense to pelvic muscles on T1W and hyperintense on T2W images with areas of necrosis; both intraspinal and extraspinal components cause widening of the sacral spinal canal and sacral foramina. T2W coronal image (C) shows clubbed, finger-like extensions along the path of the sacral nerves. Axial T1W contrast-enhanced image (D) shows intense enhancement with areas of low intensity within, corresponding to necrotic areas. Involvement of the pyriformis (arrow) and right erector spinae muscles (arrowhead) can be seen

Radiographs of the pelvis and lumbosacral spine revealed posterior scalloping of the sacral vertebrae and widened ill-defined neural foramina; there was an overlying right-sided soft tissue haze. MRI of the lumbosacral spine revealed a lobulated soft tissue mass with both intraspinal and extraspinal components in the sacral region (S2–S4). It was isointense to pelvic muscles on T1W and hyperintense, with areas of necrosis, on T2W images [Figures 1 and 2]; there was intense enhancement on postcontrast scans [Figure 2D]. It had caused widening of the spinal canal and the sacral foramina and showed clubbed finger-like projections along the path of the sacral spinal nerves. There was presacral extension and infiltration of the pyriformis and right erector spinae muscles bilaterally. Based on the MRI findings, we made the diagnosis of a complex nerve sheath tumor / soft tissue sarcoma of the sacral nerve roots.

**Figure 3:**
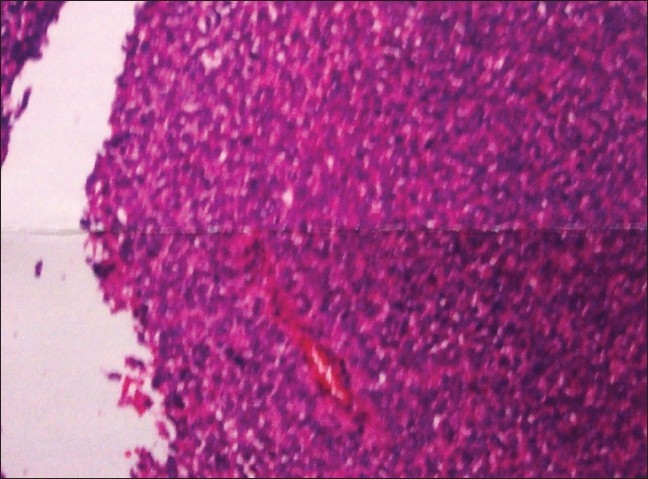
Histopathology slide (hematoxylin and eosin) shows proliferative growth of monomorphic-appearing small round cells arranged in sheets, with dispersed congested vessels. The nuclei appear round to cleaved

An open biopsy of the lesion revealed a proliferative growth. Microscopically, there were monomorphic-appearing small round cells arranged in sheets, with dispersed congested vessels. The cytoplasmic borders were indistinct, producing a syncytial appearance. Mitoses were seen. The nuclei appeared to be round to cleaved. These features are suggestive of Ewing's sarcoma / PNET [Figure 3]. Based on the clinicopathological and radiological findings, we diagnosed Ewing's sarcoma / PNET of the sacral nerve plexus.

The patient was started on chemotherapy (VAIA regimen: vincristine, adriamycin, ifosfamide, and actinomycin D), which was followed by radiotherapy to the tumor bed. Radionuclide scan of the whole body (Tc-99m-MDP) after six cycles of chemotherapy revealed no evidence of metastases. The patient responded well to the chemotherapy and radiotherapy and became asymptomatic. Repeat scans showed almost complete resolution of the mass.

## Discussion

EES / PNET is a rare malignant small round cell neoplasm of undifferentiated mesenchymal origin.[3,6] It was first described by Tefft *et al*. in 1969[7] in four patients who had paravertebral soft tissue tumors with a histologic appearance resembling Ewing's sarcoma. EES / PNET is usually seen in the second or third decades, with the reported incidence in children below 10 years being 0.5%.[6] EES / PNET has equal frequency in both males and females, as contrasted with Ewing's sarcoma of the bone (ESB), where there is a male to female ratio of 2:1.[3] The sites most commonly involved by EES / PNET are the extremities, mainly the lower limbs. Other common sites are the head and neck region, the paravertebral region, and the pelvis.[3]

In the present case, the tumor arose from the sacral spinal nerve roots; this is infrequently reported,[5] although there are reports of cases occurring in the cervical and lumbar paravertebral and epidural regions.[2–4] On MRI, the tumor is usually isointense to the muscles on T1W and hyperintense on T2W images, with enhancement on postcontrast scans, as in our case.[8] The tumor in our patient, appeared to extend along and follow the path of the sacral nerves.

The classical histopathological features of ESFT consist of uniform round cells, with irregularly shaped chromatic nuclei surrounded by scanty cytoplasm. Mitotic figures may be seen. Special cellular arrangements, like rosettes or differentiations, are not often seen. The cells often show immunohistochemical positivity for various neurofilaments, CD99, and S-100.[3,5]

Ewing's sarcoma / PNET arising from the sacral nerve plexus is only rarely reported and there is a need to differentiate it from other tumors arising in this region. The benign nerve sheath tumors such as neurofibromas are well-defined lesions, with a ‘target’ appearance due to a hyperintense rim and a hypointense center on T2W and contrast-enhanced T1W images (due to collagen and condensed Schwann cells)[9]; they are also often associated with neurofibromatosis-1. Schwannomas (neurilemmoma / neurinoma) are well-encapsulated, slow-growing benign nerve sheath neoplasms seen most commonly in the lumbar region, pushing the cord, conus, or hilum to the contralateral side; these tumors have a cystic component (40% cases) and are seen as central low-signal foci with an enhancing periphery on postcontrast T1W images.[9] Spinal ependymomas (myxopapillary) usually involve the filum terminale / conus medullaris and there is symmetric cord expansion and cavitation; these are hypointense on T1W images and hyperintense on T2W images. Hypointensity of the tumor margin on T2W images, is a sign suggestive of, but not pathognomonic of, ependymomas.[9] Non-neuraxis neoplasms such as lymphomas (particularly non-Hodgkin's lymphoma) present in older age-groups (peak incidence 40–60 years) and spread via the subarachnoid space, causing diffuse rope-like thickening of the spinal nerve roots. These lesions are often missed on MRI without contrast enhancement, as they appear isointense to the spinal cord on precontrast images. They, however show marked enhancement on postcontrast fat-suppressed MRI images.[9] Differentiation of these lesions requires imaging studies along with histopathological confirmation.[9] MRI imaging is considered the imaging modality of choice, being better than CT scan for demonstrating the pattern of tumor extension and for delineating the soft tissues.[8]

Depending on the site of the tumor and its extension, treatment can be with surgery, chemotherapy, and radiotherapy, used separately or in various combinations.[3,4] Our patient responded favorably to chemotherapy and radiotherapy.

In conclusion, the present case highlights a rare case of EES / PNET presenting in the first decade of life and arising from the sacral nerve plexus. The mass was seen to grow along the sacral nerves and the diagnosis could he established only after a biopsy. The complaints resolved following therapy.
